# Lignin-Mediated Silver Nanoparticle Synthesis for Photocatalytic Degradation of Reactive Yellow 4G and In Vitro Assessment of Antioxidant, Antidiabetic, and Antibacterial Activities

**DOI:** 10.3390/polym14030648

**Published:** 2022-02-08

**Authors:** Rijuta Ganesh Saratale, Si-Kyung Cho, Ganesh Dattatraya Saratale, Avinash Ashok Kadam, Gajanan Sampatrao Ghodake, Verjesh Kumar Magotra, Manu Kumar, Ram Naresh Bharagava, Sunita Varjani, Ramasubba Reddy Palem, Sikandar I. Mulla, Dong-Su Kim, Han-Seung Shin

**Affiliations:** 1 Research Institute of Biotechnology and Medical Converged Science, Dongguk University-Seoul, Ilsandong-gu, Goyang-si 10326, Korea; rijutaganesh@gmail.com (R.G.S.); avikadam2010@gmail.com (A.A.K.); 2 Department of Biological and Environmental Science, Dongguk University-Seoul, Ilsandong-gu, Goyang-si 10326, Korea; sk.cho@dongguk.edu (S.-K.C.); ghodakegs@gmail.com (G.S.G.); 3 Department of Food Science and Biotechnology, Dongguk University-Seoul, Ilsandong-gu, Goyang-si 10326, Korea; gdsaratale@dongguk.edu; 4 Nano Information Technology Academy, Dongguk University-Seoul, Jung-Gu, Seoul 100715, Korea; birju.srm@gmail.com; 5 Department of Life Science, Dongguk University-Seoul, 32 Dongguk-ro, Ilsandong-gu, Goyang-si 10326, Korea; manukumar007@gmail.com; 6 Department of Environmental Microbiology, School for Environmental Sciences, Babasaheb Bhimrao Ambedkar University (A Central University), Lucknow 226 025, India; bharagavarnbbau11@gmail.com; 7 Gujarat Pollution Control Board, Gandhinagar 382 010, India; drsvs18@gmail.com; 8 Department of Medical Biotechnology, Dongguk University Biomedical, Campus 32, Seoul 10326, Korea; palemsubbareddy@gmail.com; 9 Department of Biochemistry, School of Applied Sciences, REVA University, Bangalore 560 064, India; sikandar.mulla@gmail.com; 10 Department of Environmental Science and Engineering, Ewha Womans University, Seoul 120750, Korea; dongsu@ewha.ac.kr

**Keywords:** sodium lignosulfonate, silver nanoparticles (Ag NPs), photocatalytic activity, Reactive Yellow 4G, DPPH, α-glucosidase activity, antibacterial activity

## Abstract

This study explored the potential of abundantly available sodium lignosulfonate (LS) as a reducer and fabricating agent in preparing silver nanoparticles (LS–Ag NPs). The operational conditions were optimized to make the synthesis process simpler, rapid, and eco-friendly. The prepared LS–Ag NPs were analyzed via UV–Vis spectroscopy, X-ray diffraction spectroscopy, Fourier transform infrared spectroscopy, and high-resolution transmission electron microscopy. Results demonstrated that LS–Ag NPs were of crystalline structure, capped with LS constituents, and spherical in shape with a size of approximately 20 nm. Under optimized conditions, LS–Ag NPs exhibited significant photocatalytic activity in Reactive Yellow 4G degradation. The effects of photocatalyst (LS–Ag NPs) dosage, dye concentration, and its reusability for dye degradation were studied to make the process practically applicable in textile wastewater treatment. Additionally, the synthesized LS–Ag NPs displayed significant free radical scavenging against 2-diphenyl-1-picrylhydrazyl (DPPH) with an IC_50_ value of (50.2 ± 0.70 µg/mL) and also exhibited antidiabetic activity in terms of inhibition in the activity of carbohydrate-degrading marker enzyme α-glucosidase with an IC_50_ value of (58.1 ± 0.65 µg/mL). LS–Ag NPs showed substantial antibacterial potential against pathogenic strains, namely *E. coli* and *S. aureus*. In conclusion, LS–Ag NPs can be a reliable and eco-friendly material for their possible application in the treatment of dye-containing wastewater and have a great perspective in the biomedical and pharmaceutical sectors.

## 1. Introduction

Over the past years, consideration of nanomaterials has expanded substantially for numerous applications. At the end of the 20th century, nanotechnology was perceived as the next game-changer [1,2]. Green chemistry methods are essential for the progress of these imminent nanosized materials relative to chemical and physical methods. This nanotechnology approach to designing nontoxic, eco-friendly, and sustainable nanoparticles has enormous applications in all fields, including material sciences, biomedical sectors, pharmaceuticals, electronics, energy, and the environment [3]. Lignocellulosic biomass is mainly composed of three chemical constituents: cellulose (about 38–50%), hemicellulose (about 23–32%), and lignin (about 12–25%) [4,5]. The presence of lignin makes the plant cell wall more rigid, with higher mechanical strength, making it crucial to hydrolyze the cellulose and hemicellulose components. Worldwide, lignin is the most abundant renewable and biodegradable natural resource, comprising a huge quantity of aromatic groups. Thus, it is the leading research direction in the field of renewable resources [6]. Natural lignin is renewable, biodegradable, and nontoxic and has a spatial configuration with an enormous number of active groups, for instance, *p*–coumaryl alcohol, *p*–hydroxyphenyl propanol, coniferyl alcohol, sinapyl alcohol, and also various hydroxyl and aldehyde groups [4,7,8,9].

Lignin is primarily applied in lignosulfonate, which is obtained during the sulfite pulping process. Sulfonation between the sulfite solution and the lignin of the lignocellulosic biomass during sulfite pulp production increases its hydrophilicity. Further, hydrolysis of LS in acidic cooking liquid will depolymerize hemicellulose combined with lignin, enabling the separation of the lignin, cellulose, and hemicellulose, and pulp formation [6,10,11]. The physicochemical properties of LS and the details of pretreatment conditions have been mentioned in Table 1. LS has been extensively used as a dispersing agent for pesticides, dyes, and oil, as flocculating agents for water purification, and as a concrete water-reducer due to its certain advantageous physicochemical properties, as well as its being abundantly available, inexpensive, and eco-friendly [12,13]. Moreover, LS has been studied for metal NPs and metal–organic NP synthesis, for example LS-mediated ZnO NPs as an antibacterial agent; LS-fabricated Ag NPs for the removal of dyes; NaLS/SiO_2_ composite sphere particles for drug delivery; LS-synthesized Ag NPs for heavy-metal sensing, and also for electrocatalytic applications [14,15,16,17,18,19].

Conversely, the application of LS has some limitations because of lower purity after isolation, and some structural alterations in lignin molecules were observed [1]. Ag NPs are precious-metal nanomaterials with excellent electrical conductivity and a comparatively lower cost, and thus are widely utilized in various electronics, optics, catalysis, biomedical, and commercial sectors. Various physicochemical methods, including sonochemical, electrochemical, thermal decomposition, and colloidal methods are extensively studied for the synthesis of Ag NPs. However, the utilization of toxic chemical agents and their stability are limiting factors and also make the process costly and environmentally unsafe [1,2]. The green synthesis of Ag NPs, by employing plant extract, algae extract, waste biomass resources, and biopolymers as fabricating and stabilizing agents, is widely studied and also assessed for various applications [20]. The advantageous surface-modification properties of Ag NPs largely depend on the sizes, shapes, and chemical environment of Ag NPs [21,22]. Using natural pollution-free biomass materials such as LS as reducing agents and stabilizers increases its utilization value. This approach reduces environmental pollution and widens the application scope of synthesized Ag NPs.

In the present situation worldwide, more attention is engaged on wastewater pollution, instigated by rising industrialization. Textile effluent is an important pollution source in the pollution of the water environment. It was reported that approximately 17–20% of industrial water pollution originates from dyes used in the textile, dyeing, and printing industries. In the case of reactive textile dyes, approximately 50% of dyes are lost in the effluent during the dyeing process. Dye-containing wastewater also consists of various toxic contaminants, recalcitrant organics, auxiliary chemicals, surfactants, and chlorinated compounds with higher COD and BOD. Due to this, it becomes resistant to degradation, and if discharged without any treatment, these effluents will threaten the ecosystem and human life [23,24]. Therefore, dyeing effluent is essentially treated before its final discharge into the environment. Various physical, chemical, and biological approaches have been improved to treat dye-containing wastewater [24]. Among these, photocatalysis is an attractive process where the photocatalyst engrosses the light energy and consequently oxidizes the contaminants; thus, it can be a vital solution in terms of energy and environmental challenges. This process has many advantageous properties, including being inexpensive and rapid, and it can degrade whole contaminants with nontoxic, degraded by-products [25,26]. *Tribulus terrestris* extract synthesized Ag NPs, cellulose-mediated Ag NPs, selenium nanorods utilizing polyanionic cellulose, and gelatin-stabilizing Ag/Ag_2_O-NPs were extensively studied for the photocatalytic degradation of various textile dyes [27,28,29,30] (Vinay S.P., Chandrasekhar., 2019; Hamidi, et al., 2019; Vijayakumar et al., 2019; Nasab et al., 2020). Worldwide, 100 million people are affected by “diabetes,” elevated blood glucose levels. This condition causes problems in the eyes, kidneys, feet, nerves, heart, and brain vessels [31,32]. Hence, it is imperative to find medicine to inhibit carbohydrate-hydrolyzing enzymes without side effects. It was observed that under hyperglycemic conditions, there is an elevated level of reactive oxygen species that cause cellular damage and β-cell dysfunction, which results in long-term diabetic disorders [32,33].

In this work, LS was exploited as a reducing and stabilizing agent to prepare LS–Ag NPs through a simplistic and green one-pot method. Under optimal operational conditions, LS and AgNO_3_ concentrations were systematically investigated to achieve the desired properties of LS–Ag NPs, and they were further characterized using various analytical tools. Reactive Yellow 4G was designated as the model textile dye for the assessment of the photocatalytic activity of a developed LS–Ag NPs + H_2_O_2_ system in pursuance of UV-irradiation. Various operational conditions were optimized, and repeated use of the photocatalyst (LS–Ag NPs) was studied. Lastly, the in vitro biological activities concerning the antioxidant, antidiabetic, and antibacterial potential of LS–Ag NPs were studied.

## 2. Materials and Methods

### 2.1. Reagents and Chemicals

Sodium lignosulfonate, silver nitrate, 2-diphenyl-1-picryhydrazyl (DPPH), catechol, acarbose, α-glucosidase, and hydrogen peroxide (H_2_O_2_, ≥30%) were acquired from Sigma-Aldrich, St. Louis, MO, USA. All additional reagents and chemicals utilized for experiments were analytically pure and did not require further processing. Distilled water was used for solution preparation (Millipore Corporate, Billerica, MA, USA).

### 2.2. Preparation of Ls–Ag NPs

Different concentrations of sodium lignosulfonate (LS; 0.2 mM to 2.0 mM) were prepared in distilled water and placed in a 250 mL Erlenmeyer flask. The LS solution was thoroughly mixed under magnetic force for 15 min at room temperature. For the preparation of the Ag NPs, the LS solution was mixed with silver nitrate solution (1 mM) and then reacted for 3 h at 60 °C under shaking conditions (200 rpm). In favor of the property and analytical excellence of LS–Ag NPs, first, LS concentration (0.2 to 2.0 mM) was optimized by keeping the concentration of AgNO_3_ (1.0 mM) constant. Further, the effect of AgNO_3_ concentration (0.5, 1.0, and 2.0 mM) by keeping LS concentration 1.6 mM constant was studied. The color of the reaction mixture was transformed from pale yellowish to dark brown. The progression of NP synthesis was assessed by taking their absorption spectrum of solution at a function of time using UV–Vis absorption spectroscopy. Under optimized conditions, the synthesized LS–Ag NP solution was freeze-dried to obtain Ag NPs in powder form. The resultant LS–Ag NP pellet was eroded with distilled water to remove any scum and dried out in an oven (60 °C) for physicochemical characterization and in vitro biological activities.

### 2.3. Characterization of LS–Ag NPs

The physicochemical and morphological features of synthesized LS–Ag NPs were determined by using advanced analytical techniques, such as UV–Visible spectroscopy, XRD, FTIR, and HRTEM. The optical characteristics of LS–Ag NPs were assessed in the conventional and typical range of wavelength (200 to 700 nm) by the UV–Visible spectrophotometer (Optizen, Model-2120, Daejeon, Korea). The crystalline nature of biosynthesized NPs was determined by the X-ray diffraction technique. Fourier transform infrared spectroscopy was performed by a FTIR spectrometer (PerkinElmer, Norwalk, CT, USA) in the spectral range of 400–4000 cm^−1^ to identify the participation of functional groups of LS during the synthesis of LS–Ag NPs. The dimension and surface structure of the biosynthesized LS–Ag NPs were further assessed by high-resolution transmission electron microscopy (HRTEM, Tecnai G2 20 S-TWIN, FEI Company, Loughborough, UK). Size dissemination of LS–Ag NPs was inspected through the typical method stated earlier [22].

### 2.4. Photocatalytic Degradation of Reactive Yellow 4G by Synthesized LS–Ag NPs

The photocatalytic activity of synthesized LS–Ag NPs was evaluated to degrade RY4G as a model dye. In the first step, 5 mg of LS–Ag NPs were put into 100 mL of RY4G dye solution (20 mg/L) in a 150 mL beaker. The reaction solution was vigorously mixed and sonicated in the dark for 30 min for better dispersion of the catalyst LS–Ag NPs in the reaction mixture, and then 1 mL of 1 wt% H_2_O_2_ was added into the reaction mixture prior to UV-light irradiation. Afterwards, the reaction mixture was subjected to UV-light irradiation at a UVA wavelength of 365 nm, keeping a distance of about 15 cm, and an intensity of 1000 μW/cm^2^ (Vilber Lourmat multi-lamp photoreactor, Vilber, Marne-la-Vallée cedex, France). The progress of photocatalysis leads to a gradual decrease in color measured using a UV–Vis spectrophotometer as a function of time. The deprivation of dye concentration during photocatalysis was deliberated by measuring the relative diminution in absorbance at *λ*_max_ of RY4G. Moreover, the photocatalytic ability of LS–Ag NPs was studied at different concentrations of RY4G (10, 20, 30, and 40 mg/L), keeping constant 10 mg of LS–Ag NP concentration, and the effects of increasing photocatalyst concentration of LS–Ag NPs (5, 10, and 15 mg/L) against the RY4G maintaining its concentration constant at (20 mg/L). Finally, the reusability of the photocatalyst (LS–Ag NPs) concerning the degradation of RY4G (20 mg/L) was studied under optimized conditions [34]. In this study, the reaction mixture without photocatalyst was considered a control. All photocatalytic degradation examinations were executed in triplicate.

### 2.5. In Vitro Biological Activities (Antidiabetic, Antioxidant, and Antibacterial) of Synthesized LS–Ag NPs

The in vitro antidiabetic prospective of LS–Ag NPs was studied by quantifying the inhibition aptitude contrary to α-glucosidase, a marker carbohydrate-hydrolyzing enzyme. The α-glucosidase assay was executed in line with the standard protocol and assessed the inhibition of the enzyme activity by LS–Ag NPs at various concentrations (20, 40, 60, 80, and 100 µg/mL) [35]. NaLS and acarbose were considered control and standard for this enzyme assay. In vitro antioxidant activity of ascorbic acid (as standard), NaLS, and produced LS–Ag NPs were explored by evaluating the free radical scavenging activity against 2,2-diphenyl-1-picrylhydrazyl (DPPH). The DPPH scavenging enzyme assay followed the standard protocol [22]. The antioxidant activity was deliberated by taking median and typical deviation values, whereas the scavenging potential was appraised using the previously described procedure.

The in vitro antibacterial potential of LS–Ag NPs was carried out against *Escherichia coli* and *Staphylococcus aureus* using typical Kirby–Bauer disc diffusion by following the methodology reported earlier [22,36]. Deionized water was reciprocated as a negative control, whereas ampicillin was considered as a positive control. The zone of inhibition and the antimicrobial index of LS–Ag NPs contrary to individual contagious bacteria was estimated and quantified [37].

### 2.6. Statistical Analysis

All the experimentations were performed in triplicate, and the outcomes of all calculated values are reflected as mean ± standard error mean (SEM). The data attained were inferred using the one-way analysis of variance (ANOVA) test convoyed by a Tukey–Kramer multiple comparisons test.

## 3. Results and Discussion

### 3.1. Synthesis of Lignosulfonate Mediated Ag NPs and Optimization of Conditions

Silver nanoparticle synthesis should be simple, technically feasible, performed without using toxic chemical-reducing agents, and have exceptional material properties. With this aim in this study, lignosulfonate was utilized for Ag NP synthesis to develop a simpler, more cost-effective, and more eco-friendly process.

**Figure 1:**
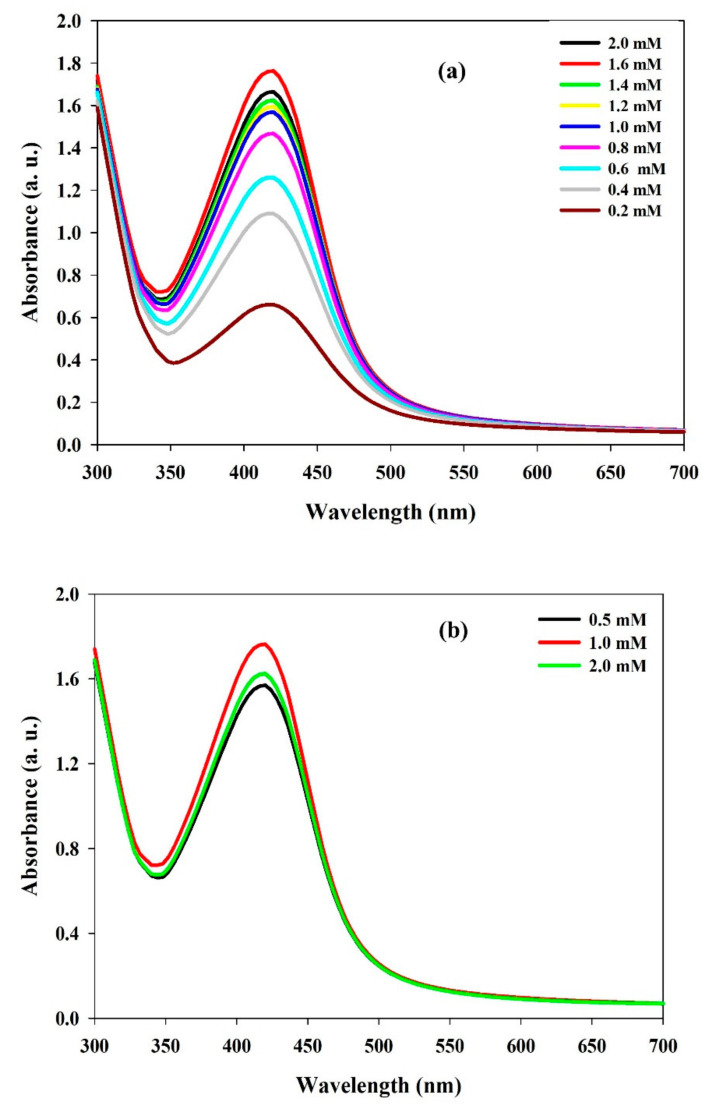
Effects of (**a**) lignosulfonate concentration (0.2 mM to 2.0 mM) and (**b**) silver nitrate concentration (0.5 to 2.0 mM) on LS–Ag NP synthesis.

First, the reaction-process conditions were optimized. The optimal reaction conditions were: reaction time (3 h), reaction temperature (60 °C), and shaking condition (200 rpm), and they were utilized in further experiments (data not shown). UV–Visible spectroscopy was used to monitor the biosynthetic and eco-friendly reduction process between LS and silver salts. The absorbance vs. wavelength curve of the nanoparticles was measured and confirmed by a UV–Visible spectrophotometer in the typical wavelength range of 200 to 700 nm. The typical outstanding absorption maxima peak confirmed the reduction of Ag^+^ ions to Ag^0^ and the formation of LS–Ag NPs at 418 nm (Figure 1a). Our findings agree with other Ag NPs synthesized using lignosulfonate molecules, where the synthesized Ag NPs exhibited typical SPR peaks at 410–420 nm [15,17,38].

Optimization of the ratio between LS and silver salt is essential since it impacts the synthesis of NPs, morphology, and characteristics. Initially, the effects of increasing LS concentration (0.2 mM to 2.0 mM) by keeping AgNO_3_ (1 mM) concentration constant was studied. The results showed that, with increasing LS concentration, there is an upsurge in the intensity of the SPR peak, which directly illustrates that the amount of Ag NP synthesis becomes amplified (Figure 1a). Therefore, it was supposed that an increase in LS concentration enhanced the nucleation process rate, by which there is an increase in LS–Ag NP synthesis taking place up to the saturation rate. In our study, up to 1.6 mM of LS concentration, an increase in SPR peak was observed; however, further increase in LS concentration diminished the SPR peak (Figure 1a).

**Figure 2:**
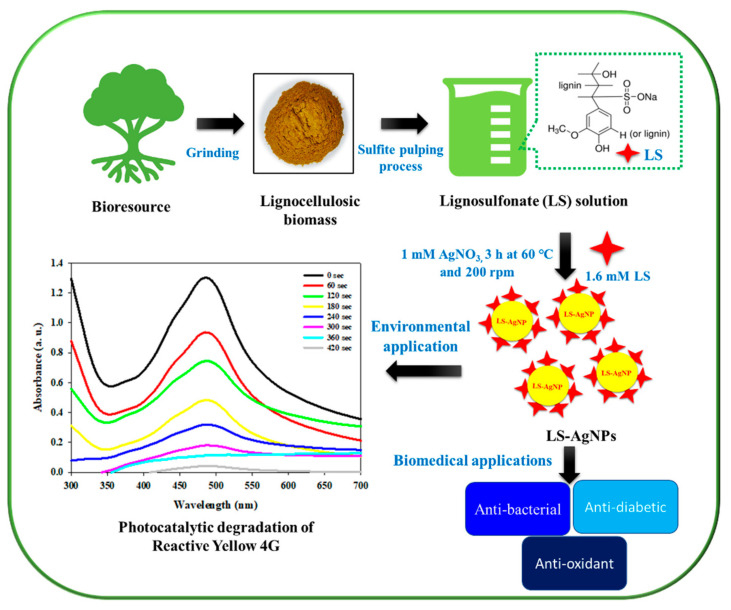
Schematic representation of the proposed research work.

Similarly, we studied the effects of AgNO_3_ concentration on NP synthesis using UV–Vis analysis. The results showed an upsurge in SPR intensity peak with AgNO_3_ concentration. Figure 1b displays the UV–Vis spectra of LS–Ag NPs produced from dissimilar AgNO_3_ concentrations. The maximum intensity of the SPR peak was observed at 1.0 mM AgNO_3_ concentration. However, further increasing the AgNO_3_ concentration, a decline in the SPR peak was recorded (Figure 1b). This might be due to the agglomeration of produced LS–Ag NPs. The results also suggest that silver nitrate concentration significantly influenced Ag NP formation. In our synthesis process, well-defined SPR peaks were observed without broadening the absorbance peak. The results signified that the particle-size distribution of LS–Ag NPs is very narrow and spherical in shape. Similar results were observed in other lignosulfonate-mediated Ag NP synthesis studies [17,38]. The foregoing results suggest that 1.6 mM of LS and 1 mM of AgNO_3_ are optimal concentrations for significant LS–Ag NP production. The schematic representation of the research work is depicted in Figure 2.

### 3.2. Analytical Characterization of Synthesized LS–Ag NPs

#### 3.2.1. XRD Analysis

**Figure 3:**
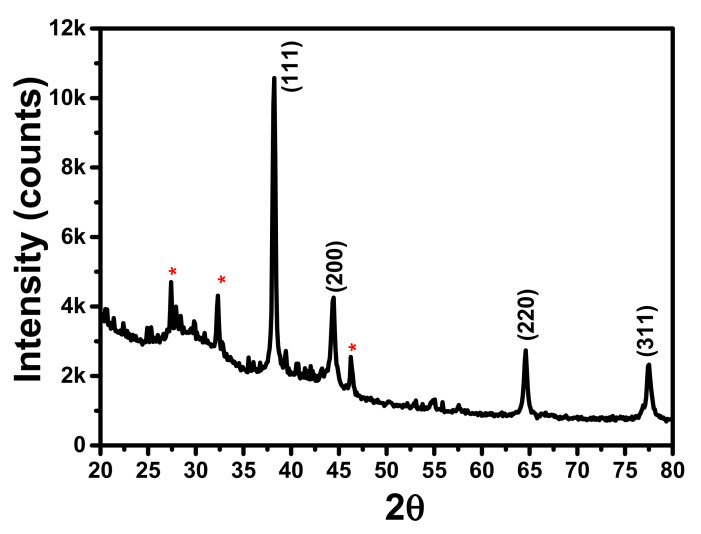
XRD pattern of LS–Ag NPs synthesized under optimized conditions where minor undesignated peaks (denoted with stars).

X-ray powder diffraction (XRD) is a rapid analytical technique used to determine the crystalline nature and phase identification of synthesized NPs. The XRD pattern investigation exhibited that the LS–Ag NPs were found to be crystalline. In Figure 3, peaks present at 38.2^0^, 44.4^0^, 64.6^0^, and 77.5^0^ of the 2θ correspond to the (111), (200), (220), and (311) planes of cubic face-centered silver (JCPDS file No. 5-2872). Similar peaks were observed in other studies of Ag NPs synthesized using lignin molecules [15,17,39]. The average particle size of Ag NPs was assessed by using the Debye Scherrer formula (d *= 0.9λ*/*(*β cos θ), where β is full-width half maxima (FWHM) of the XRD peak, λ is the wavelength of the X-ray Cu Kα source (1.54 Å), and θ is the Bragg diffraction angle. The average size of the LS–Ag NPs was found to be 22.9 nm, which is in agreement with the average size determined by HRTEM analysis. The lattice constant calculated from LS–Ag NP XRD data was found to be ‘*a*’ = 4.07 Å. The microstrain (ε) value was calculated by the formula (microstrain (ε) = β/4 tanϴ). The microstrain was found to be 1.44 × 10^−3^ for Ag NPs. Minor undesignated few peaks (denoted with stars) were also detected, signifying that the crystallization of bioorganic phase develops on the exterior surface of LS–Ag NPs [40,41,42].

#### 3.2.2. FT-IR Analysis

**Figure 4:**
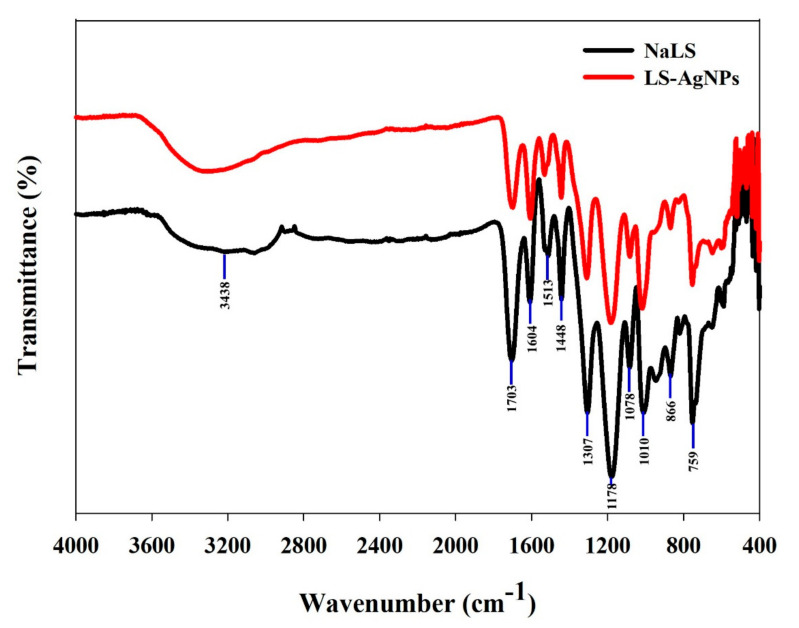
FTIR analysis of LS–Ag NPs synthesized under optimized conditions.

FTIR spectroscopy in the spectral range of 400 to 4000 cm^−1^ was performed to know about the possible involvement of different functional groups of LS in the reduction process of the silver ions to nanoparticles. The broad absorption spectra, between 3200–3400 cm^−1^ of LS and LS–Ag NPs, are the characteristic peaks of phenolic hydroxyl groups [43]. The absorption peaks at 1703 cm^−1^ and 1604 cm^−1^ were ascribed to the stretching of C=O in carboxylic acid and its derivatives, whereas the 1510 cm^−1^ peak is related to the benzene skeleton vibration [44,45]. The characteristic peak at 1448 cm^−1^ is assigned to the aromatic ring in the lignosulfonate. Moreover, the peaks at 1078 and 1178 cm^−1^ correspond to the sulfonic groups (-SO^3−^ and S=O) of LS, respectively [15]. Additionally, the absorption peaks in the range of 1000 and 800 cm^−1^ were related to aromatic vibrations groups of C=O, C-O, and C-H [3]. The whole topography is shown in Figure 4. The sodium lignosulfonate contains carboxyl, carbonyls, phenolic, and aliphatic hydroxyl functional groups, all of which were shown to be involved in the production of nanoparticles. According to our findings, these functional groups participated in reducing silver ions to LS–Ag NPs. These findings are in line with silver nanoparticles synthesized using different biomolecules [3,15,45].

#### 3.2.3. HR-TEM Analysis

**Figure 5:**
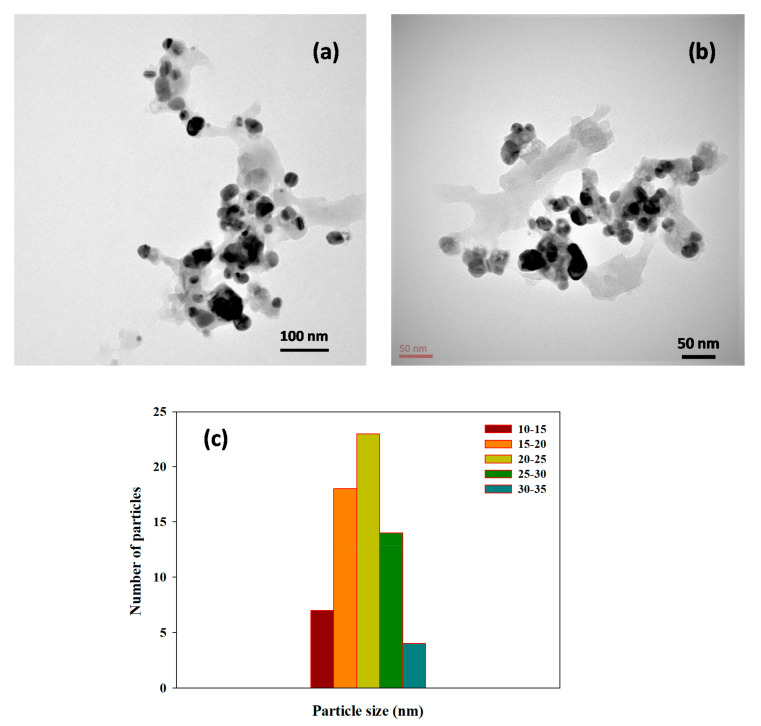
HR-TEM images of LS–Ag NPs: (**a**) at 100 nm; (**b**) at 50 nm amplification; and (**c**) average particle-size histogram of the LS–Ag NPs produced under optimized conditions.

Electron microscopy is a useful analytical technique for analyzing the dimension and exterior surface of the produced nanoparticles. HR-TEM was used to investigate the physical dimensions and morphological properties of LS–Ag NPs. TEM pictures of LS–Ag NPs at a different magnification of 100 nm to 50 nm displayed that the produced LS–Ag NPs are spherical and monodispersed, uniform, and consistently dispersed in the sample (Figure 5a,b), which are in line with the XRD and UV–Visible spectroscopy results.

The presence of dark spots on the exterior surface of the LS–Ag NPs indicates the fabrication of NPs with LS chemical constituents. The particle histogram was analyzed from each TEM image, and the average size of LS–Ag NPs was calculated in the range of 15 to 25 nm (Figure 5c). Similar interpretations were documented in the Ag NPs synthesized using *Acacia nilotica* leaf extract, tannic acid, and *Dracocephalum kotschyi* aqueous extract [3,46,47].

### 3.3. Photocatalytic Degradation of RY4G Using LS–Ag NPs and Optimization of Reaction Conditions

**Figure 6:**
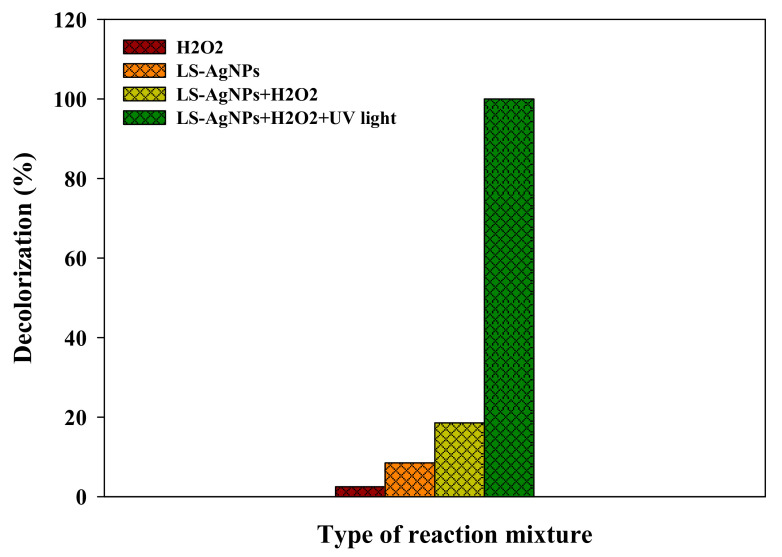
The effect of the redox mediator and UV-light irradiation on the photocatalytic degradation of Reactive Yellow 4G by LS–Ag NPs.

RY4G has been selected as a model dye to assess the photocatalytic activity of LS–Ag NPs with H_2_O_2_ as a redox mediator and in the presence of UV irradiation. To understand the effect of each factor in the preliminary investigation, the photocatalytic activity of (a) only LS–Ag NPs, (b) only H_2_O_2_, (c) LS–Ag NPs + H_2_O_2_ under dark conditions, and (d) LS–Ag NPs + H_2_O_2_ + UV irradiation was systematically investigated. The results are displayed in Figure 6. Only H_2_O_2_ and LS–Ag NPs were found to be ineffective in the degradation of RY4G and resulted in 2.5 and 8.5% deprivation of dye content. At the same time, LS–Ag NPs + H_2_O_2_ was also found less effective and led to 18.5% degradation of dye molecules. This might be due to the adsorption of dye on the catalyst and slight degradation in the presence of H_2_O_2_. However, in the presence of UV irradiation and LS–Ag NPs + H_2_O_2_ system showed complete photocatalytic degradation of RY4G. The results indicate that LS–Ag NPs + H_2_O_2_ and UV-light irradiation are responsible for the complete degradation of dye. After degradation of dye, there is no change in color of the photocatalyst, which suggests that the LS–Ag NP catalyst is stable, and no dye is adsorbed on the exterior surface.

**Figure 7:**
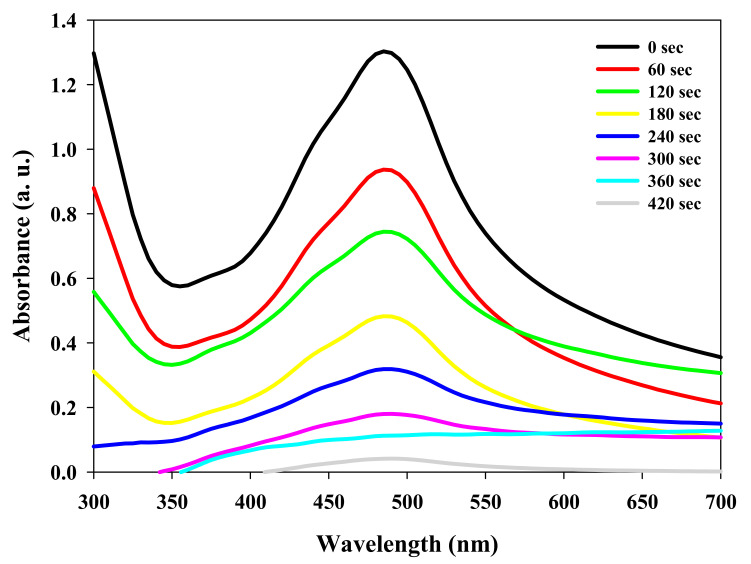
Time course photocatalytic degradation of Reactive Yellow 4G by developed LS–Ag NPs + H_2_O_2_ system under UV-light irradiation.

It was supposed that the aromatic structures of lignosulfonate decomposed the H_2_O_2_ to discharge more active radicals (·OH and ·HO_2_) while interacting with UV light. Furthermore, due to the surfactant properties of LS, it induces the affinity of LS–Ag NPs towards dye molecules. The results also indicate that developed LS–Ag NPs have exceptional dispersion and distribution, increasing the contact area for effective interaction with dye molecules [15,48]. These consequences lead to the complete degradation of dye molecules by the developed LS–Ag NPs + H_2_O_2_ system under UV-light irradiation. During the time course of photocatalytic degradation of RY4G by LS–Ag NPs + H_2_O_2_ system under UV-light irradiation, there was a decline in the absorption peak intensity at 485 nm. Complete elimination of peak was observed after 6 min (Figure 7), which indicates degradation of RY4G by the developed photocatalytic system [3,49]. Similar observations were made in other studies related to the photocatalytic degradation of textile dyes [50,51].

**Figure 8:**
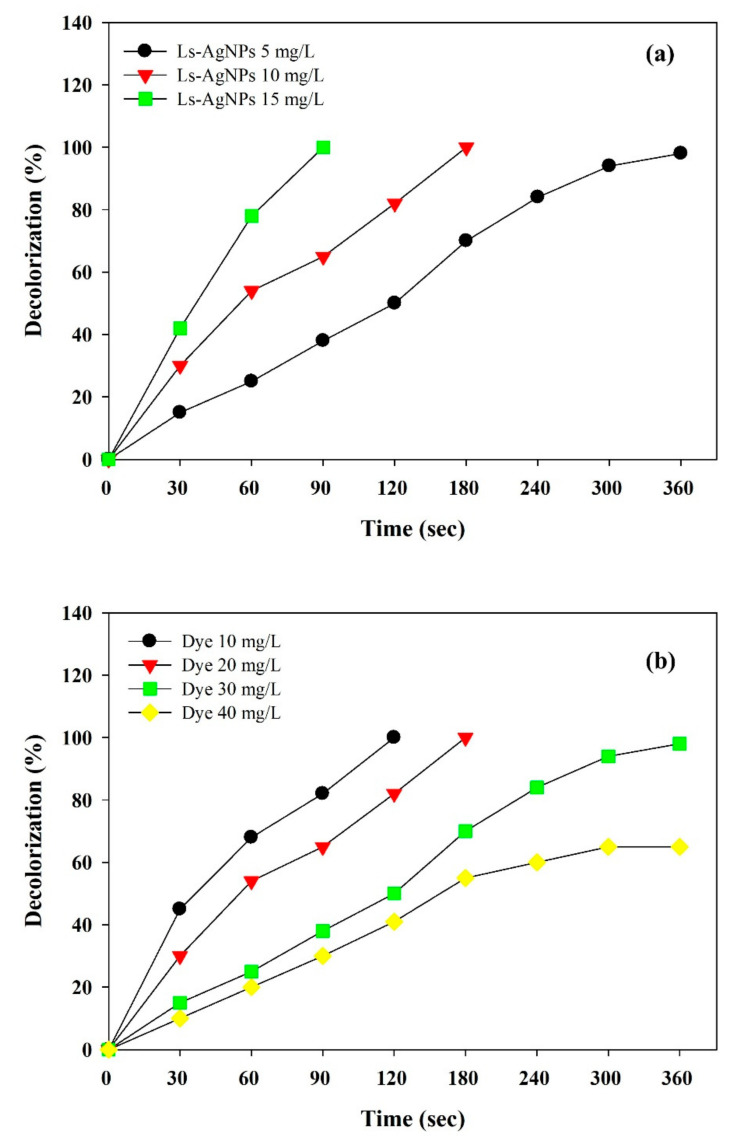
Effects of increasing (**a**) photocatalyst dosage and (**b**) dye concentration on the photocatalytic degradation of Reactive Yellow 4G by developed LS–Ag NPs + H_2_O_2_ system under UV-light irradiation.

Optimization of photocatalyst dosage and dye concentration are vital factors in developing the photocatalytic process and achieving better performance. Further, the effects of increasing the photocatalyst by keeping RY4G concentration (20 mg/L) and increasing dye concentration by keeping LS–Ag NP concentration (10 mg/L) constant. The results are presented in Figure 8a,b. The time (360 s) required for complete deprivation of RYG4 by LS–Ag NPs is relatively lower than that in other photocatalytic studies employing green-synthesized Ag NPs, including *Convolvulus arvensis* leaf extract mediated Ag NPs for the degradation of Reactive Black 5, Methyl Orange; Direct Yellow-142 required 60 min, whereas *Eriobotrya japonica* (Thunb.) leaf extract Ag NPs for the degradation Reactive Red 120 and Reactive Black 5 required 30 min using sodium borohydride as a redox mediator [31,52].

### 3.4. Recyclability of LS–Ag NPs

**Figure 9:**
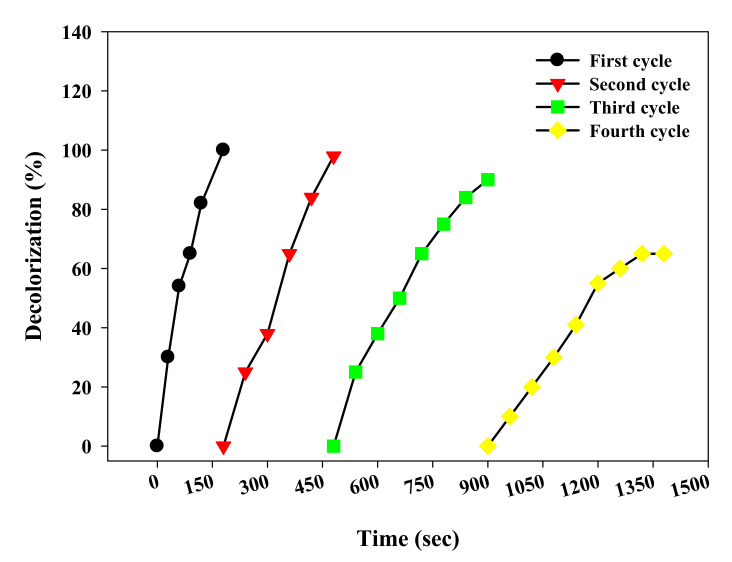
Repeated use of developed LS–Ag NPs + H_2_O_2_ system on the photocatalytic degradation of Reactive Yellow 4G under UV-light irradiation.

The catalyst reusability, stability, and lifespan are important factors to develop in the photocatalytic process for long-term applications. In the repeated use of LS–Ag NPs, study photocatalyst concentration (LS–Ag NPs 10 mg/L) and dye concentration 20 mg/L) were employed under optimized conditions. The results suggest that LS–Ag NPs showed significant photocatalytic activity up to the third cycle, with complete dye degradation (Figure 9). Further increase in the cycle increases time (600 and 720 s) for complete degradation of dye up to the fourth and fifth cycle, respectively.

The increase in time during recyclability of LS–Ag NPs might be due to weight loss of LS–Ag NPs during the retrieval procedure, aggregation of the catalyst particles, intervention of the light by the suspension, and scattering properties leading to a decrease in the diffusion of photons [53,54,55]. However, the results are noteworthy and increase their applicability for treating dye-containing wastewater.

### 3.5. In Vitro Antioxidant and Antidiabetic Activity of Synthesized LS–Ag NPs

**Figure 10:**
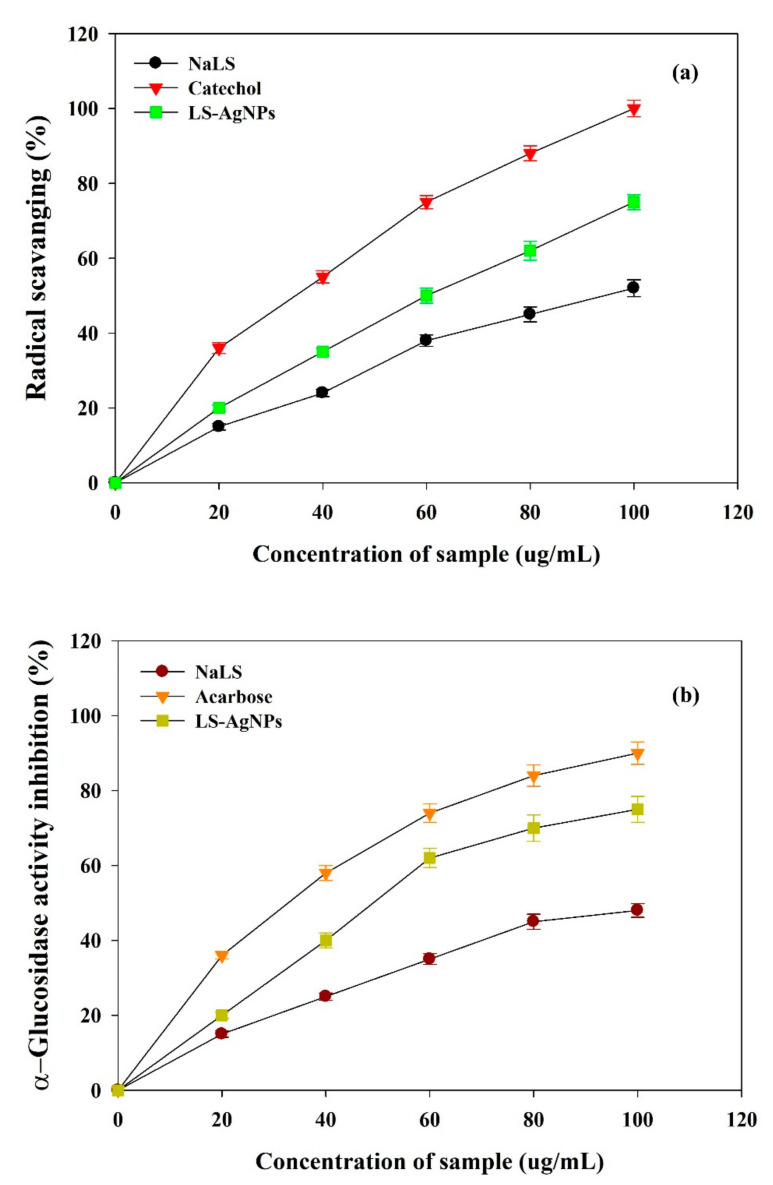
(**a**) Antioxidant potential in terms of scavenging activity against highly stable DPPH; and (**b**) antidiabetic potential (inhibition) against α-glucosidase by synthesized LS–Ag NPs.

Traditional medicine usage increased globally due to existing modern synthetic drugs’ side effects during the last decade. Antioxidants are known for their medical benefits, including anticancer and antidiabetic activities. In addition, they help remove free radicals, protect cells from injury, and reduce inflammation. The DPPH free radical scavenging experiment was performed to test the antioxidant property of the LS–Ag NP nanoparticles. This antioxidant assay is a well-known test that can accurately determine the antioxidant capacity of any given chemical compound or nanoparticle. LS–Ag NPs showed significant activity and successfully scavenged DPPH free radicals up to 75.2 ± 2.12 at 100 µg/mL, which is substantially higher than only NaLS 52.1 ± 1.15 at 100 µg/mL (Figure 10a). The IC_50_ value of LS–Ag NPs and catechol for DPPH scavenging were 34.5 ± 0.78 µg/mL and 50.2 ± 0.70 µg/mL, respectively. The obtained antioxidant activity is substantially higher than other lignin-capped Ag NPs [56,57].

α-glucosidase is an enzyme responsible for the degradation or conversion of carbohydrates into glucose and thus plays a vital role in controlling glucose levels. High glucose levels lead to severe clinical complications; therefore, a way should be found to control glucose levels. Enzyme inhibition is an antidiabetic activity.

For that purpose, different concentrations of LS–Ag NPs (20 to 100 µg/mL) were prepared and checked against the α-glucosidase enzyme for evaluating the in vitro antidiabetic activity. LS–Ag NPs showed dose-reliant inhibition of α-glucosidase enzyme activity with half-inhibitory concentration IC_50_ of 58.1 ± 0.65 µg/mL (Figure 10b). The results of enzyme inhibition by standard acarbose and LS are shown in Figure. The literature suggests the potent antidiabetic activities of green-synthesized Ag NPs, and our results follow their results [58,59,60]. These results increase the potential applications of LS–Ag NPs for biomedical applications; however, more research is still required by in vivo studies, which will be our future research viewpoint.

#### In Vitro Antibacterial Studies

Moreover, due to the excess use of antibiotics, multidrug-resistant bacterial strains are rising worldwide, alarming to human health. This also underlines the necessity to develop effective antimicrobial agents [61]. When it comes to antibacterial activities, size and concentration are important factors. Smaller sizes may readily pass through bacterial protective barriers and enter deep into the microorganism to do more significant harm. The antibacterial properties of LS–Ag NPs against pathogenic bacteria, namely *E. coli* and *S. aureus,* were deliberated by calculating the zone of inhibition. Individual sodium lignosulfonate (LS) has shown no significant antibacterial activity against the test microorganisms. The results are presented in Table 2.

LS–Ag NPs showed a significant antibacterial index with standard antibiotic ampicillin relative to selected bacterial strains. Nanoparticles’ antibacterial activity is due to their interactions with, and disruption of, cell membranes, causing the release of intracellular materials. The foregoing results suggest that the synthesized LS–Ag NPs execute substantial antibacterial activity due to the presence of the phenolic components of lignin and thus can be used to develop antibacterial drugs. In line with the results, significant antibacterial activity by lignosulfonate incorporated into chitosan nanoparticles and lignin-fabricated Ag NPs have been reported [62].

### 3.6. Advantages of the LS–Ag NPs and Future Research Perspectives

The utilization of waste lignosulfonate generated during the sulfite pulping process for Ag NP synthesis is novel, simple, environmentally benign since there is no use of toxic chemicals during synthesis, and cost-effective. The developed LS–Ag NPs-H_2_O_2_ system in the presence of UV irradiation gave satisfactory photocatalytic degradation of RY4G and the repeated use of LS–Ag NPs as a photocatalyst, which increases its potential applicability for textile wastewater treatment. Further, research should be devoted towards reactor development and its implementation for actual wastewater treatment, its mineralization, and toxicity of degraded products. In the preliminary investigation, synthesized LS–Ag NPs showed significant in vitro antioxidant, antidiabetic, and antibacterial activities; however, more research is still required by in vivo studies, which will be our future research viewpoint.

## 4. Conclusions

Utilization of lignosulfonate derived from the pulping process for nanoparticle synthesis is imperative and has economic and environmental benefits. LS acts as a reductant and stabilizer to prepare nanomaterials and conforms to the concept of green chemistry. The developed LS–Ag NPs found an ideal photocatalyst and showed significant photocatalytic degradation of Reactive Yellow 4G under optimized conditions. Results demonstrated that LS–Ag NP photocatalytic activity is stable and can be repetitively utilized three times, increasing their practical applicability. This study provides a new idea for the practice of green chemistry and a novel method for dye-wastewater treatment. Further, synthesized LS–Ag NPs showed significant antioxidant, antidiabetic, and antibacterial activity, which is noteworthy. We believe that the synthesis of LS–Ag NPs is rapid, cost-effective, and eco-friendly, and thus increases their feasibility for diverse applications, including environmentally and biomedically oriented research fields.

## Figures and Tables

**Table 1:** Chemical compositions, properties, and treatment conditions of lignosulfonate (LS) adapted from [1,8,9].

Parameter	Lignosulfonate
Treatment conditions	Metal sulfite + sulfur dioxide (Ca^2+^, Mg^2+^ or Na^+^) (pH = 2–12, T = 120–180 °C, for 1–5 h)
Solubility	Water
Ash content (mass %)	4.0–9.3
Sulfur (%)	3.5–8.0
Carbohydrates (mass %)	ND
Molecular weight (Da)	1000–50,000
Polydispersity Index (PDI)	4.2–8.0

**Table 2:** Antimicrobial activity of Ta-Ag NPs against pathogenic microorganisms *E. coli* and *S. aureus*.

	Zone of Inhibition (mm)			
Bacteria Strain	LS–Ag NPs (20 μg/mL)	Ampicillin (20 μg/mL)	Sodium Lignosulfonate (20 μg/mL)	Antimicrobial Index (%)
*Escherichia coli*	15.8 ± 0.38	16.8 ± 0.35	4.45 ± 0.45	94.0 ± 2.45
*Staphylococcus aureus*	12.2 ± 0.54	14.2 ± 0.41	3.25 ± 0.23	85.9 ± 2.98

Ampicillin: positive control; Sodium lignosulfonate: negative control. Values are mean ± standard error of three replicates.
